# Selecting variant masks to improve power and replicability of gene-level burden tests

**DOI:** 10.21203/rs.3.rs-6322956/v1

**Published:** 2025-04-15

**Authors:** Trang Nguyen, Ryan Koesterer, Sean J. Jurgens, Peter Dornbos, Satoshi Yoshiji, Alex Llamas, Dongkeun Jang, Patrick Smadbeck, Annie Moriondo, Quy Hoang, Oliver Ruebenacker, Patrick Ellinor, Noël Burtt, Jason Flannick

**Affiliations:** 1 Program in Medical & Population Genetics, The Broad Institute of MIT and Harvard, Cambridge, MA, USA; 2 Cardiovascular Disease Initiative, The Broad Institute of MIT and Harvard, Cambridge, MA, USA; 3 Department of Experimental Cardiology, Heart Center, Amsterdam Cardiovascular Sciences, Heart Failure and Arrhythmias, Amsterdam UMC location University of Amsterdam, Amsterdam, The Netherlands; 4 Cardiovascular Research Center, Massachusetts General Hospital, Harvard Medical School, Boston, MA, USA; 5 Regeneron Genetics Center, Tarrytown, New York, USA; 6 Division of Genetics and Genomics, Boston Children’s Hospital, Boston, MA, USA; 7 Program in Metabolism, The Broad Institute of MIT and Harvard, Cambridge, MA, USA; 8 Department of Pediatrics, Harvard Medical School, Boston, MA, USA; 9 Department of Human Genetics, McGill University, Montréal, Québec, Canada; 10 Lady Davis Institute, Jewish General Hospital, McGill University, Montréal, Québec, Canada; 11 Canada Excellence Research Chair in Genomic Medicine, Victor Phillip Dahdaleh Institute of Genomic Medicine, McGill University, Montréal, Québec, Canada; 12 Kyoto-McGill International Collaborative Program in Genomic Medicine, Graduate School of Medicine, Kyoto University, Kyoto, Japan; 13 Precision Cardiology Laboratory, The Broad Institute of MIT and Harvard, Cambridge, MA, USA; 14 Cardiology Division, Massachusetts General Hospital, Boston, MA, USA

## Abstract

Rare coding variant association studies typically perform gene-level association tests in which variants are filtered (or “masked”) and aggregated based on functional annotation and allele frequency. As there is little research and no consensus regarding masking strategies to use, we investigated the impact of masking strategies on gene-level burden tests, the most widely used and interpretable type of aggregate association test. A systematic review of 234 studies catalogued 664 masks and masking strategies that rarely repeated across studies. Analyzing 54 traits within 189,947 UK Biobank exomes, we show that the number of significant associations greatly depends on the masking strategy employed (ranging from 58 to 2,523 associations) and, consequently, separate published analyses of this dataset report minimally overlapping associations (<30%). By empirically determining mask combinations that maximize the number of significant associations, we propose masking strategies that detect twice as many significant low-frequency and rare variant associations as the “average” strategies previously employed, with consistent performance across many traits. Our analyses demonstrate the inconsistency of previously used variant masking strategies and provide a simple solution to increase power and replicability in future studies.

## Introduction

Coding variant gene-level association tests – in which variants are grouped within a gene and tested for aggregate association with a trait – are central to whole exome sequencing (WES) and whole genome sequencing (WGS) association studies^1–3^. Various gene-level testing methodologies have been developed^2^, each of which uses different assumptions to estimate the association between the grouped variants and a phenotype. Some tests, like the Sequence Kernel Association Test (SKAT)^4^ or SKAT-O^5^, use advanced approaches to maintain power when variants have different or even opposite effect sizes^5^. Many, if not most studies, however, perform burden tests^6,7^ – which are particularly useful for drug discovery^8,9^ and functional follow-up^3^ due to their simplicity and interpretability. Because burden tests assume that all included variants have the same impact on gene function, selecting the variants included in the test is especially important^10^.

The simplest and most common type of variants included in a burden test are putative loss-of-function (pLoF) variants^11,12^, as these have the highest probability of disrupting gene function and can be predicted with high accuracy^13^. Theory suggests, however, that including missense alleles can triple effective sample size – but only if disruptive missense alleles can be distinguished from benign ones^10^. Experimental approaches for identifying disruptive missense alleles have been shown to significantly improve the power of burden tests^12–14^, but they can only be applied to genes with established experimental assays.

By contrast, variant filters based on frequency and bioinformatic annotations (e.g., PolyPhen2^15^, CADD^16^) can be applied to any gene. Early WES studies defined four variant “masks” according to which of five bioinformatic algorithms predicted a missense variant as damaging^17,18^. These studies then employed a “masking strategy” in which four gene-level association tests – one per mask – were performed for each gene. More recently, as the number of bioinformatic prediction algorithms has increased^19^ and as databases of variant effect predictions have become available^20^, the number of potential masking strategies has increased substantially^21^. Nonetheless, these potential strategies have not yet been incorporated into standard practice for WES or WGS studies, as demonstrated by recent high-profile studies^22,23^. Therefore, there remains little clarity as to how different masking strategies affect the results of gene-level burden tests and a lack of guidelines as to how to incorporate these strategies into a gene-level analysis.

In this study, we investigated (a) how masking strategies used in previous studies varied in their ability to detect gene-level burden associations and (b) if certain masking strategies could consistently increase the number of significant associations (following multiple test correction for the number of masks in the strategy). We addressed these questions by applying burden tests to 54 quantitative traits within whole exome sequences from 189,947 UK Biobank participants^24^. Our results demonstrate substantial variability across previously employed masking strategies and suggest three masking strategies to increase burden test power and replicability for future studies. Based on the results, we developed a script for filtering variants based on the recommended strategies (github.com/broadinstitute/genemasker) and applied them to 54 continuous traits in 439,829 exome samples from the UK Biobank^24^ (hugeamp.org:8000/research.html?pageid=trang_mask_paper_home).

## Results

### Previous masking strategies employed in coding variant association studies

**Figure 1.**
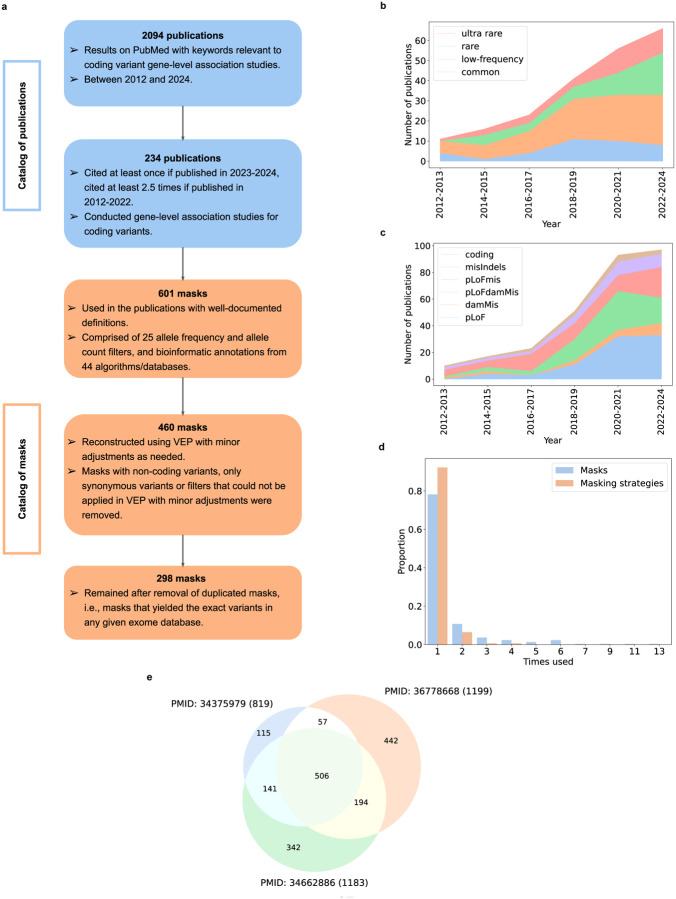
Summary of previously employed masks and masking strategies. **(a)** Summary of the review process of gene-level association studies and mask usage. **(b)** Number of publications that employed the four maximum minor allele frequency (maxMAF) thresholds over the years (**Supplementary Table 3**). From top to bottom: ultra rare (red), rare (green), low-frequency (orange), common (blue). **(c)** Number of publications that employed six types of bioinformatic annotations over the years (**Supplementary Table 3**). From top to bottom: coding (brown), misIndels (purple), pLoFmis (red), pLoFdamMis (green), damMis (orange), pLoF (blue). **(d)** Usage frequency of masks and masking strategies after mask harmonization in VEP. **(e)** Overlap of significant associations between three high-profile studies of the UK Biobank (after Bonferroni correction) for 46 continuous phenotypes: PMID: 34375979 (~470K samples), PMID: 36778668 (~500K samples), and PMID: 34662886 (~455K samples).

We surveyed mask usage within the literature from 2012 to 2024 by searching PubMed using keywords for coding variant association analyses and then filtering results for citation rates and content (Fig. 1a; **Supplementary Table 1;** Methods), identifying 234 studies that had performed gene-level association tests. These collectively used 664 masks (**Supplementary Table 2a)**, 601 of which had clearly documented definitions. These 601 clearly documented masks employed bioinformatic annotations from 44 algorithms/databases and 25 allele frequency/count filters (Fig. 1a; **Supplementary Table 2b**). Since many masks differed solely due to implementation details (e.g., annotation tools or reference databases), we harmonized them within a common software pipeline using the Variant Effect Predictor (VEP)^25^, dbNSFP^26^, and UK Biobank frequency filters, slightly altering mask definitions as needed (Methods). We also removed 141 masks that contained non-coding variants, contained only synonymous variants (often used as negative controls), or could not be easily implemented using VEP^25^ and dbNSFP^26^ (Methods). These filters resulted in 460 masks, 298 of which were unique (Fig. 1a; **Supplementary Tables 2c-d**). These masks were applied as part of 154 unique masking strategies within 169 publications.

The 460 masks grouped broadly into 24 categories, defined by 6 bioinformatic annotation types and 4 maximum MAF (maxMAF) thresholds (**Supplementary Tables 2a, 3**). Low-frequency masks (maxMAF < 1%) appeared the most (92 studies, 183 masks), with rare (maxMAF < 0.1%) and ultra-rare (maxMAF < 0.01%) variant masks beginning to replace common variant masks (maxMAF > 1%) since 2019 (Fig. 1b). The shift towards rarer variants is consistent with increasing sample sizes of WES and WGS studies over time, making it possible to detect significant associations with variants of smaller MAF. In terms of bioinformatic annotations, pLoF variants (83 studies, 116 masks), and pLoF or damaging missense variants (pLoFdamMis; 71 studies, 150 masks) have remained the two most popular types (Fig. 1c). Perhaps surprisingly, some studies exclude pLoF variants to only focus on medium-impact variants^27–29^ (**Supplementary Table 2a**).

Strikingly, 78.2% of masks and 92.2% of masking strategies were used in only one publication (Fig. 1d). This if anything understates the lack of consistency in mask usage across studies, as without mask harmonization the non-repeated fractions were even larger (91.7% of masks and 98.5% of masking strategies). A notable example of the impact of different mask usage on association results was provided by three recent high-profile studies of the UK Biobank (PMID: 34662886^22^, PMID: 36778668^23^, PMID: 34375979^30^) that employed, respectively, a 10-mask strategy (pLoF variants across five MAF thresholds, pLoF or missense variants predicted to be damaging by five bioinformatic algorithms across five MAF thresholds), a 2-mask strategy (pLoF variants with MAF < 1%, missense / indel / low-confidence pLoF variants with MAF < 1%), and a 9-mask strategy (combinations of protein-truncating variants, missense variants predicted to be damaging by REVEL^31^ and non-synonymous variants with varying MAF thresholds) (**Supplementary Table 2a;** Methods). Despite each study analyzing the same exome dataset (between ~455K and ~500K samples of the UK Biobank), only 506 (28.2%) out of 1,797 significant associations across the three studies (for 46 continuous traits; Methods) were shared by all three and 898 (50%) by at least two (Fig. 1e).

### Variability in associations across previously employed masks and masking strategies

**Figure 2.**
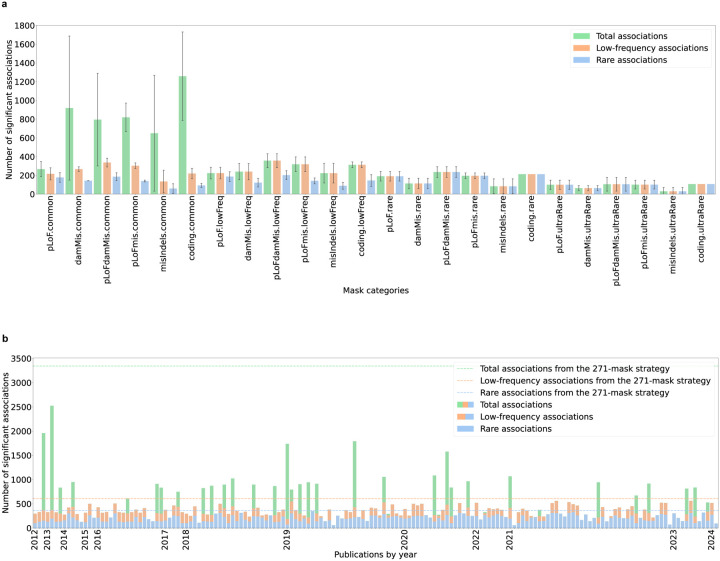
Number of significant associations for 54 phenotypes across 24 mask categories and 163 publications. **(a)** Average number of total (green), low-frequency (orange) and rare (blue) significant associations in 24 mask categories (**Supplementary Tables 3, 7a**). Error bars represent standard deviations. **(b)** Number of total (green + orange + blue), low-frequency (orange + blue) and rare (blue) significant associations detected by the masking strategy employed in each publication (**Supplementary Table 8**). Dashed lines represent the number of total (green), low-frequency (orange) and rare (blue) significant associations produced by the 271-mask strategy.

To examine more fully the extent to which differences in mask definition led to differences in association results, we conducted gene-level burden tests for each of the 298 masks across 55 quantitative traits and 17,605,686 variants within 189,947 (~190K) UK Biobank whole exome sequences (we used this subset of the full 439,829 exome dataset due to its offline availability; **Supplementary Table 4;** Methods). We excluded poorly calibrated analyses (6 masks across all traits and all masks for height due to exceptionally high genomic inflation) and low-count genes and masks, after which 271 masks (used in 163 studies) and 54 traits remained for analysis (**Supplementary Tables 5a-b, 6;** Methods). As a negative control, burden tests of synonymous variants with MAF < 0.1% yielded only 7 significant (*P* < 2.5 × 10^−6^) associations across 54 traits. By contrast, the other masks collectively produced 6,563 significant associations. The number of significant associations varied widely by masks, ranging from 3 (for a mask consisting of missense variants predicted to be damaging by PolyPhen2 with MAF < 0.001%^32^) to 2,706 associations (for a mask consisting of pLoF variants / missense variants predicted to be damaging by SIFT^33^ or PolyPhen2^15,34^; **Supplementary Fig. 1a; Supplementary Table 7a**). This pattern of wide variation across masks was also observed for nearly every trait individually (**Supplementary Fig. 1b**). As expected, variation in the number of significant associations was greater between masks in different categories than it was between masks in the same category, although there was still substantial variability within each of the 24 categories (F-statistic = 22.27, *P* = 1.23 × 10^−47^ for all associations; Fig. 2a).

Notably, a large fraction of these associations resulted from masks including common variants, which due to linkage disequilibrium (LD) are more likely than rare variants to be tagging causal variants outside of the mask (**Supplementary Table 7a**). We conducted a conditional analysis including covariates for common variants associated with each trait and found that the number of significant associations reduced by 23.7% (SD = 22.5%; **Supplementary Fig. 2a; Supplementary Table 7b;** Methods). Since most WES studies aim to explore low-frequency and rare variant associations, we conducted two further analyses to filter the significant associations to those detectable via only low-frequency variants (MAF < 1%; low-frequency associations) or rare variants (MAF < 0.1%; rare associations) (Methods). The number of low-frequency and rare significant associations still varied widely across different masks, albeit to a lesser extent compared to total significant associations for all traits combined (ranging from 3 to 440 and 3 to 289, respectively) and nearly every trait individually (**Supplementary Figs. 1a, 1c-d; Supplementary Table 7a**), with similar patterns of variability within and between mask categories (F-statistic = 23.26, *P* = 3.75 × 10^−49^ for low-frequency associations; F-statistic = 13.73, *P* = 2.05 × 10^−32^ for rare associations; Fig. 2a). We note that, as previously demonstrated^35^, LD between variants contributing to these associations and common variants was much reduced but not fully eliminated (**Supplementary Figs. 2b-c; Supplementary Table 7b**) – conditional analysis is needed to demonstrate complete independence of rare significant associations from common SNPs.

The number of significant associations across the 146 masking strategies previously employed in 163 studies showed even greater variation than it did across the 271 previously employed masks (Fig. 2b; **Supplementary Table 8**). Across the 54 traits, the number of significant associations (after Bonferroni correction for the number of masks in the strategy) ranged from 58 (for a two-mask strategy from PMID: 35108381^36^ aggregating pLoF singletons or missense singletons) to 2,523 (for a two-mask strategy from PMID: 23633568^34^ aggregating pLoF and damaging missense variants with MAF < 1% or with no MAF restriction), with slightly less variability for low-frequency (58 to 563 associations) and rare significant associations (58 to 352 associations) (Fig. 2b; **Supplementary Tables 2a, 8**). There was no clear trend in the number of significant associations versus the year in which the masking strategy was defined, suggesting that masking strategies have not necessarily become more powerful over time (Fig. 2b; **Supplementary Table 8**).

### Potential masking strategies to increase the number of significant associations

A seemingly naive (but comprehensive) masking strategy would be to run association tests for every mask ever defined. This “brute-force” strategy, even after Bonferroni correction for 271 masks, produced 7.3-fold more total, 1.9-fold more low-frequency, and 1.8-fold more rare significant associations than the “average” strategies previously employed (3,344 total, 605 low-frequency, and 361 rare; Fig. 2b; **Supplementary Table 8;** Methods). The “best” masking strategies previously employed detected 75.4% as many total significant associations (PMID: 23633568^34^), 93.1% as many low-frequency significant associations (PMID: 37558884^9^), and 97.5% as many rare significant associations (PMID: 31383942^37^) as did the 271-mask strategy (**Supplementary Table 8;** Methods). These results suggest that it should be possible to increase the power of gene-level burden tests – compared to previous studies – through a more judicious choice of masking strategies.

**Figure 3.**
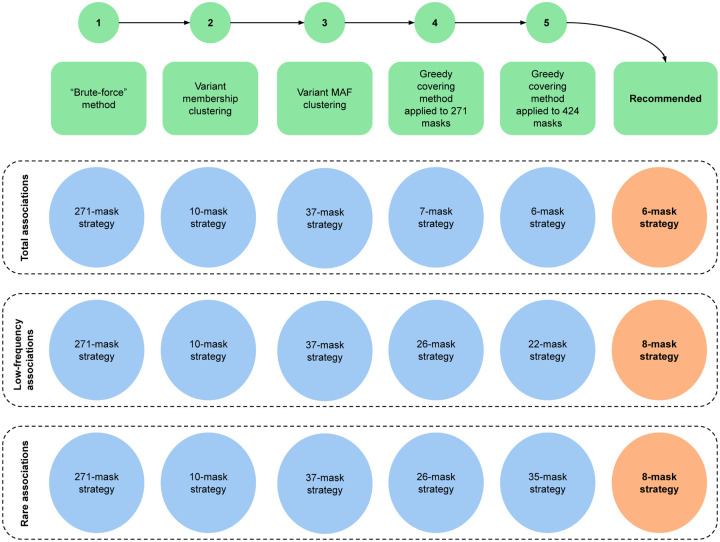
Workflow of analysis to obtain recommended masking strategies. **(1)** We considered the 271-mask strategy as a brute-force method. **(2)** We applied PCA on the binary inclusion of variants in 271 masks and conducted k-means clustering on the first 9 PCs. This resulted in 10 clusters, each of which was “represented” by the mask with the largest number of total, low-frequency or rare significant associations; these masks made up the 10-mask strategies. **(3)** Among the 10 clusters, PCA and k-means were applied again to variant MAF, dividing the 10 clusters into 37 subclusters. The mask with the largest number of significant associations (total, low-frequency, rare) represented each subcluster, resulting in 37-mask strategies. **(4)** The greedy covering method iteratively selected the mask with the largest number of additional significant associations after Bonferroni correction for each number of masks out of 271 masks. **(5)** The greedy covering method was applied to the new set of 424 masks to produce masking strategies with the maximum number of significant associations. All the methods were applied to total associations, low-frequency associations and rare associations separately. Finally, we recommended three masking strategies that consisted of 6 or 8 masks that could detect at least 95% of the maximum number of total, low-frequency or rare significant associations. See Methods for full details.

To improve upon the brute-force 271-mask strategy, we first attempted to identify redundant masks by applying Principal Component Analysis (PCA)^38^, using the binary inclusion of UK Biobank variants as features (variant membership clustering; Fig. 3; Methods). The first 9 principal components (PCs) explained 90% of the total variance of the 271 masks, but clustering based on the first 9 PCs produced 10 overlapping groups of masks with substantial heterogeneity within each cluster (silhouette score = 0.51; **Supplementary Figs. 3a-b; Supplementary Table 9a**). To reduce this heterogeneity, within each cluster we performed a second round of PCA using the UK Biobank variants’ MAFs as features (variant MAF clustering; Fig. 3; Methods). Subdividing each cluster based on its first few PCs produced a total of 37 comparatively homogeneous subclusters (mean of silhouette scores = 0.79, SD = 0.12; **Supplementary Table 9b**). This analysis suggests that the “effective number” of independent masks previously employed in the literature is closer to 40.

**Figure 4.**
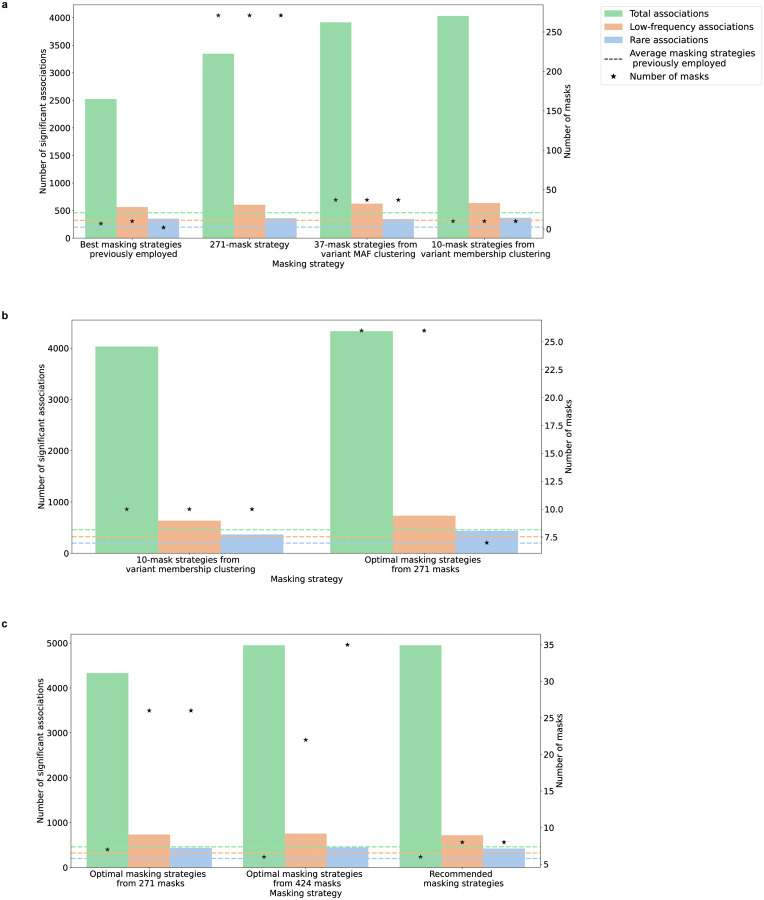
Number of significant associations produced by various masking strategies (after Bonferroni correction for the number of masks). Green bars represent total associations, orange bars low-frequency associations, blue bars rare associations. Green dashed lines represent total associations, orange dashed lines low-frequency associations, blue dashed lines rare associations by average masking strategies previously employed. Stars represent the number of masks in each masking strategy. The figure shows the number of significant associations produced by **(a)** best masking strategies previously employed, the brute-forced 271-mask strategy, 37-mask strategies from variant MAF clustering, and 10-mask strategies from variant membership clustering **(b)** 10-mask strategies from variant membership clustering and optimal masking strategies from 271 masks, **(c)** optimal masking strategies from 271 masks, optimal masking strategies from 424 masks, and recommended masking strategies. See **Supplementary Table 10** and Methods for more details.

We hypothesized that selecting one “representative” mask from each cluster might identify a similar number of significant associations as the 271-mask strategy. We constructed three 10-mask and three 37-mask strategies in which we selected the mask that produced the most total, low-frequency, or rare significant associations within each cluster or each subcluster (Fig. 3; **Supplementary Tables 9c-d;** Methods). After Bonferroni correction, the 10-mask strategies from variant membership clustering each produced more total, low-frequency, and rare significant associations than did the 271-mask strategy, and they produced substantially more associations than the average masking strategies previously employed (Fig. 4a; **Supplementary Table 10**). However, the 37-mask strategies from variant MAF clustering produced slightly fewer significant associations than did the 10-mask strategies. This decrease in performance was due to the cost of increased Bonferroni correction outweighing the benefit of adding more masks. These results suggest that much of the benefit of the 271-masking strategy can be achieved with a much smaller number of masks, but mask clustering may not produce the “optimal” strategy.

We therefore attempted to directly optimize the number of significant associations produced by a masking strategy, using a “greedy covering” method in which we iteratively added masks to the strategy based on the number of additional significant associations identified across the 54 traits (Fig. 3; Methods). We validated the robustness of this approach via a cross-validation analysis in which we selected masks based on 53 traits and evaluated the significant associations identified for the remaining trait (**Supplementary Fig. 4; Supplementary Table 11;** Methods). This greedy approach yielded “optimal” 7-mask, 26-mask, and 26-mask strategies for total, low-frequency, and rare significant associations (**Supplementary Figs. 5a-c; Supplementary Tables 10, 12a**). The optimal masking strategies produced 301 more total significant associations (7.5% increase), 97 more low-frequency significant associations (15.3% increase), and 70 more rare significant associations (19.1% increase) than did the 10-mask strategies from variant membership clustering (Fig. 4b; **Supplementary Table 10**). Notably, the number of masks in the optimal strategy for total associations was substantially smaller than the number for low-frequency and rare associations. This is because most common variant associations (but not rare variant associations) are captured by a small number of masks, and adding more masks only removes significant associations due to Bonferroni correction (Methods). The optimal masking strategy therefore depends on the frequency of associations that one desires to detect.

### Potential improvements from new masks

As our analysis to this point was restricted to masks previously employed in the literature, we next asked if we could define additional masks to further increase burden test power. We derived two types of new masks: (a) 117 masks from applying stricter MAF thresholds to the 271 previously employed masks; and (b) 36 masks combining pLoF variants with missense variants predicted to be damaging by different numbers of bioinformatic algorithms (**Supplementary Table 6**; Methods). Across the 54 traits we analyzed, the 424 (= 271 + 117 + 36) masks collectively produced (ignoring Bonferroni correction) 969 more significant associations than the 271 masks. We repeated the greedy covering method for the expanded set of masks and obtained three new optimal 6-mask, 22-mask, and 35-mask strategies for total, low-frequency, and rare significant associations (**Supplementary Table 12b**). The 6-mask total strategy exceeded its 7-mask counterpart by 14.3% (619 significant associations, with 809 gained and 190 lost; **Supplementary Table 10**). Among the gained associations, 639 were not significant in any mask of the 7-mask strategy and 170 did not survive 7-mask Bonferroni correction. However, there were smaller increases in the number of low-frequency (23 associations, 3.1% increase) and rare (8 associations, 1.8%) significant associations produced by the optimal 22-mask and 35-mask strategies compared to their 26-mask counterparts (**Supplementary Table 10)**.

### Recommended masking strategies

In summary, the optimal 6-mask (total), 22-mask (low-frequency), and 35-mask (rare) strategies identified substantially more significant associations, relative to any masking strategy previously employed in the literature, across the 54 traits we analyzed. While the truly optimal set of masks for any study is unknowable, our results suggest these strategies to be relatively robust across different traits compared to an average strategy from the literature (mean of proportion = 10.7, SD = 5 for total significant associations, mean of proportion = 2.1, SD = 0.8 for low-frequency significant associations; mean of proportion = 2, SD = 1.1 for rare significant associations; **Supplementary Figs. 6a-c**). In the event that the 22-mask and 35-mask strategies are too large for a researcher to apply in practice, our analyses indicate that the gain in significant low-frequency and rare associations attenuates rapidly after 8 masks, which captured approximately 95% as many associations as did the full strategies and twice as did the average masking strategies previously employed (Fig. 3; Fig. 4c; **Supplementary Table 10**). Like the larger strategies, the smaller 8-mask strategies also show consistent numbers of significant associations produced across traits (mean of proportion = 1.9, SD = 0.8 for low-frequency significant associations; mean of proportion = 1.8, SD = 0.8 for rare significant associations; **Supplementary Figs. 6b-c**). Depending on interest in total, low-frequency, or rare significant associations, a sensible starting point for future gene-level burden analyses may therefore be the 6-mask (total), 8-mask (low-frequency), or 8-mask (rare) strategies (Table 1).

As an example, when applied to 54 traits in ~190K UK Biobank exomes, the recommended 8-mask strategies detected 166 low-frequency and 64 rare significant associations not found by any of the 19 masks (harmonized in VEP) used by the three previous high-profile studies of this same dataset^22,23,30^. When we compared the number detected by the 8-mask strategies to the number detected by the masking strategies employed in those high-profile studies (after Bonferroni correction), the increase was higher: between 273–285 more low-frequency associations and between 106–125 more rare associations. An even more substantial number of additional significant associations were identified by the 6-mask strategy (between 4,519 and 4,625 more associations). However, most (between 93% and 94.8%) of the gained associations were driven by common variants and likely detectable by variant-level association studies such as GWAS. Therefore, we focused our subsequent analyses on the recommended low-frequency and rare 8-mask strategies.

To validate the significant associations detected by the low-frequency and rare 8-mask strategies, we conducted several analyses of the 166 low-frequency and 64 rare significant associations (202 unique associations) detected by the two 8-mask strategies but not by any mask from the three studies^22,23,30^ (**Supplementary Table 13a**). First, of these 202 associations, 179 were within a significant GWAS locus (**Supplementary Table 13b**), providing strong prior evidence that they represent true associations. Second, when applied to the larger set of 439,829 (~440K) samples in the UK Biobank^24^, significance increased for 186 out of 230 associations; an additional eight associations remained exome-wide significant (*P* < 2.5 × 10^−6^) despite decreasing in significance. Finally, we conducted gene-set enrichment analyses (across 4,203 gene sets from the Mouse Genomic Informatics database^39^) on the burden results from the ~440K samples using a previously described method^21^. Across the 54 phenotypes, we found 421 significant gene set associations for the low-frequency 8-mask strategy and 211 significant gene set associations for the rare 8-mask strategy (*P* < 0.05 after FDR correction). Most of these enrichments were for mouse phenotypes previously linked to the human traits (**Supplementary Tables 14a-b**). These results suggest that the additional associations detected by the recommended strategies are enriched for true, rather than artifactual, associations.

### Novel associations from the recommended masking strategies

The recommended rare 8-mask strategy detected 899 significant associations (after Bonferroni correction) in the extended ~440K analysis across 46 traits, 127 of which were not detected by any of the three published studies of greatly overlapping UK Biobank samples^22,23,30^ (**Supplementary Tables 15a-b;** Methods). Of these, 87 associations were significant due to the collective signal of many variants, none of which were exome-wide significant on their own. The 127 novel rare associations included genes within three categories: known “effector genes” for a trait, genes within GWAS loci for a trait but not known as an effector gene for the GWAS signal, and genes with no prior genetic links to a trait.

An example of a gene in the first category (known effector genes) is *PRG2*, a major component of the crystalline core of the eosinophil granule^40^. Several non-coding variants whose closest gene is *PRG2* were associated with eosinophil count, including rs548854 (MAF = 0.39, beta = −0.02, *P* = 8.2 × 10^−20^)^41^ and rs639509 (MAF = 0.27, beta = −0.02, *P* = 5.1 × 10^−25^)^41^. This is consistent with the eosinophil count association (EAF = 8.7 × 10^−6^, beta = −0.28, *P* = 1.1 × 10^−9^, cumulative MAC (cMAC) = 498, number of variants = 61; **Supplementary Table 15a**) we observed for the *PRG2* in a mask consisting of rare pLoF variants, indels and damaging missense variants predicted by Polyphen2 (Table 1), which suggests that reduction of *PRG2* activity decreases eosinophil count.

An example of a gene in the second category (effector gene candidates) is *PTPN11*, the coding gene for SHP2 protein^42^, which was associated with hemoglobin A1C (HbA1C) by a mask consisting of rare pLoF variants and (likely) pathogenic variants predicted by ClinVar (Table 1; EAF = 2.8 × 10^−6^, beta = −0.57, *P* = 1.5 × 10^−8^, cMAC = 98, number of variants = 38; **Supplementary Table 15a**). A recent review discussed the potential protective or promoting roles of SHP2 on insulin resistance^43^ while another study identified the over-expression of *PTPN11* in patients with type 2 diabetes complicated with colorectal cancer^44^. Common variants near *PTPN11* were also associated with HbA1C, including rs11066309 (MAF = 0.39, beta = −0.03, *P* = 3.9 × 10^−26^)^45^ and rs1029850317 (MAF = 0.33, beta = −0.02, *P* = 2.7 × 10^−18^)^45^. The rare variants have an aggregate effect size one order of magnitude larger than those observed for the non-coding variants, providing an expanded and more impactful allelic series relating *PTPN11* to HbA1C.

Another example is an association for *PLAG1*, which was found to be over-expressed in children with obesity compared to children without obesity^46^. Several animal studies demonstrated an important role of *PLAG1* in body growth^47,48^. Furthermore, non-coding variants near this gene were associated with total lean body mass (TLBM) including rs72656010 (MAF = 0.13, beta = −0.36, *P* = 8.6 × 10^−61^)^49^ and rs62515408 (MAF = 0.13, beta = −0.28, *P* = 8.4 × 10^−38^)^49^. Rare variants in *PLAG1* were associated with TLBM in a mask consisting of rare pLoF variants and damaging missense variants predicted by REVEL (Table 1; EAF = 1.2 × 10^−6^, beta = −1, *P* = 4.1 × 10^−8^, cMAC = 12, number of variants = 11; **Supplementary Table 15a**), further consistent with the role of *PLAG1* in body growth.

Finally, an example from the third category (novel genes) includes *PTPN6*, a member of the protein-tyrosine phosphatases family, which was found to participate in platelet signal transduction^50^. *PTPN6* plays an important role in signaling pathways of hematopoietic cells^51^ and inflammatory processes^52^. In mice, PTPN6 was found to inhibit platelet apoptosis and necroptosis^53^; and in zebrafish, *ptpn6* knockdown was found to impair the innate immune system^54^. The rare variant association between platelet count and *PTPN6* variants in a mask consisting of rare pLoF variants and damaging missense predicted by REVEL (Table 1; EAF = 2.5 × 10^−6^, beta = −0.42, *P* = 1.9 × 10^−9^, cMAC = 184, number of variants = 81; **Supplementary Table 15a**) is consistent with the role of *PTPN6* in platelet regulation and the immune system.

A final example of a novel association in this category is for *ASXL1,* which has been studied extensively for its role in myeloid diseases^55^ and found to be responsible for BohringOpitz syndrome (BOS)^56^. Rare variants in *ASXL1* were associated with forced vital capacity by a mask consisting of rare pLoF variants and (likely) pathogenic variants predicted by ClinVar (Table 1; EAF = 5.4 × 10^−6^, beta = −0.31, *P* = 2.4 × 10^−13^, cMAC = 379, number of variants = 75; **Supplementary Table 15a**). Supporting the validity of this association, a 2018 study found that *Asxl1* ablation in mice caused defective lung maturation^57^, and abnormal alveoli formation was associated with *ASXL1* variants in an autopsy of a newborn who died of BOS^58^.

These results demonstrate that, not only does the recommended rare 8-mask strategy produce substantially more significant rare associations than previously employed masking strategies, but the novel associations are also enriched for true associations and have a high level of biological plausibility. All results from this analysis are publicly accessible via a web browser (hugeamp.org:8000/research.html?pageid=trang_mask_paper_home), and we also developed a publicly available script for defining and constructing the recommended masking strategies according to a user-specified list of variant sites (github.com/broadinstitute/genemasker).

## Discussion

While gene-level association studies, particularly WES association studies, have produced many important discoveries^22,23,30^, the lack of standard protocols for variant filtering and grouping – a part of nearly every WES study – limits the consistency and reproducibility of gene-level associations. Our results demonstrate this lack of consistency by cataloguing a large number of masks and masking strategies employed in previous studies that have never been used again. Our results suggest this lack of consistency reduces the number of significant associations that could be detected by an intelligent selection of masks. By applying these masking strategies to 54 quantitative traits across UK Biobank 189,947 exome sequences, we recommend three simple masking strategies consisting of 6 or 8 masks that should immediately increase the number of significant total, low-frequency and rare variant associations by 10.8-fold, 2.2-fold and 2.1-fold compared to average masking strategies previously employed (**Supplementary Table 10**). These results appear to hold across a wide variety of traits with different genetic architectures (**Supplementary Fig. 6**).

Our study has several limitations. First, we designed our recommended masking strategies by considering associations across a variety of traits within a single dataset (the UK Biobank), with samples of one ancestry (European), and for one type of gene-level association test (burden test). Even though it is not possible to determine in advance whether another masking strategy would be preferable for a new study, the relatively consistent performance of the masks across traits is encouraging (**Supplementary Fig. 6**). Furthermore, unlike common variants whose genetic signals may differ between ancestries, groups of rare variants (MAF < 0.1%) are more likely to have similar aggregate genetic effects as other groups of rare variants with similar molecular effects, and therefore, rare variant masking is likely to be transferable across most populations^21,59^. Second, different traits have various degrees of polygenicity and heritability, which might influence the performance of any mask given and the variation across masks; however, outlier traits demonstrating unusual mask performance tend to have lower power compared to the rest of the traits (**Supplementary Figs. 1b-d; Supplementary Table 11**). Third, the greedy covering method we used for identifying the optimal masking strategies is also known to be an approximation, although it is unlikely that an exact algorithm would substantially improve upon the masking strategies we identified (Methods). Finally, beyond the number of significant associations produced, a researcher might consider other factors including false positive/negative rates, novel association discovery, and concordance or aggregation of results between masks.

Beyond any limitations of our study, researchers should also consider the broader limitations of variant aggregation tests in gene-level association studies. First, different masks tend to yield different gene-level burden statistics and sometimes even opposite directions of effect for the same gene. Our analysis indicated that this event is rare in general (5.9% of genes with multiple significant associations had opposite directions of effect between masks, none in the rare 8-mask strategy), but discordances may indicate allelic series with opposite phenotypic impacts within a gene, which may be of interest for experimental gene characterization or therapeutic development. Second, if researchers are interested in masking strategies for tests such as SKAT and SKAT-O that make less strong assumptions about variant effects, mask selection may not be as critical. We chose to focus in this study on burden tests as opposed to SKAT or SKAT-O due to their simplicity, interpretability, and widespread use, but a full analysis of masks in the context of other tests may be of future interest. Finally, our results underscore that masks rely on bioinformatic predictions of variant functional impact, as experimental estimates of variant effects are infeasible to obtain at a genome-wide scale. If, in the future, better methods become available for predicting or measuring variant impact, it may be possible to reduce the number of masks within an optimal masking strategy.

Regardless of these limitations, our study suggests several clear recommendations for authors of gene-level burden studies. First, researchers should be cognizant of the impact that a masking choice has on their results and transparently report and justify why they selected certain masks. Second, applying our recommended masking strategies is a justifiable default choice that appears to detect more associations than past strategies over a wide range of traits. Third, researchers should consider the relative value of total or low-frequency gene-level associations (which may overlap GWAS hits or even be non-causal proxies of common variant signals) as compared to rare variant associations less affected by LD. Fourth, researchers can use our script to facilitate the generation of these masking strategies, particularly for the widely used UK Biobank dataset (github.com/broadinstitute/genemasker). Finally, gene-level burden results for 54 continuous traits from the UK Biobank ~440K exome sequences using our recommended masking strategies are publicly available via a web browser (hugeamp.org:8000/research.html?pageid=trang_mask_paper_home), and may be a viable alternative to conducting association tests for many researchers who want to quickly interrogate “genetic support” for a gene of interest^60^. While there is no substitute for careful and context-specific association study design, our data-driven approach converges on three masking strategies consisting of 6 or 8 masks (no MAF threshold, MAF < 1% and MAF < 0.1%) that will increase the power and replicability of future gene-level burden studies.

## Methods

### Literature review of variant masks and masking strategies

We define a mask as a combination of filters of bioinformatic annotations, minor allele frequency (MAF) and/or minor allele count (MAC) thresholds on coding variants, which are then collapsed for a gene-level association test. On the other hand, a masking strategy is a set of masks employed in an analysis or a publication. On February 28, 2024, we searched PubMed for scientific articles published between 2012 and 2024 that employed variant masking strategies for gene-level association tests with the two sets of keywords: (1) rare variants AND (burden OR skat) and (2) “whole-exome sequencing” AND (gene-level OR gene-burden OR “coding variants”). The union of these two searches resulted in 2094 journal articles (**Supplementary Table 1**). Only papers published between 2012 and 2022 that were cited at least 2.5 times a year (609 publications) and papers published between 2023 and 2024 that were cited at least once (119 publications) were included, totaling 728 publications.

Next, we skimmed through the 728 publications and coded them regarding their relevance to coding variant gene-level association studies as follows: (0) irrelevant after title scanning (297 publications), (1) relevant to coding variant gene-level association studies, i.e., masks were used for gene-level association tests (215 publications), (2) irrelevant after skimming through the papers for various reasons such as coding variant association studies were not conducted and variants were not collapsed on the gene level (178 publications), (3) full papers not accessible with credentials from the Broad Institute of MIT and Harvard (38 publications). In addition to the 215 relevant publications, we added 19 other studies that we had identified. As a result, we shortlisted 234 publications that conducted coding variant genelevel association tests (**Supplementary Table 1)**.

### Data and samples used

For the ~190K analysis, we obtained genotypes for 200,631 consented samples from the UK Biobank^24^ (application ID: 41189) October 2020 version of the “Population level exome OQFE variants, PLINK format - interim 200k release (data field: 23155)”. For the ~440K analysis, we used the UK Biobank Research Analysis Platform (RAP) to obtain genotypes for 469,818 consented samples from the UK Biobank “Population level exome OQFE variants, * format – final release” (* = BGEN/PLINK/pVCF, data fields: 23159/23158/231257). For sample quality control in the extended data, we pre-filtered the variants of the PLINK (data field: 23158) version by removing the low-quality variants identified in the UK Biobank provided helper file “ukb23158_500k_OQFE.90pct10dp_qc_variants.txt”.

We selected 28 continuous phenotypes with different genetic architectures previously analyzed for their rare variant burden heritability^32^. We then added 27 more phenotypes in various phenotype groups (anthropometric, cardiometabolic, hematological, etc.) to increase the phenotype pool (**Supplementary Table 4**). Since UK Biobank participants had multiple visits, we took the mean of all measurements and inverse-normalized the mean values to ensure normality before association testing.

### Data quality control (QC): Variant QC

We followed the standard variant and sample QC procedures^61^. For the ~190K analysis, we downloaded a raw PLINK file set to a local computing cluster, whereas for the ~440K analysis, due to computing limitations caused by the size of the data and UK Biobank downloading restrictions, we handled the data differently as follows.

After splitting multi-allelic loci, the ~190K PLINK file set had 17,908,846 variants. We removed 303,160 variants with call rate < 0.9, heterozygosity = 1, or Hardy Weinberg equilibrium *P* < 1 × 10^−6^, leaving 17,605,686 for association testing. For the ~440K analysis, we pre-filtered the dataset using the UK Biobank provided helper file, leaving 21,500,124 variants for association testing.

To generate variants used for sample QC, we applied conservative filters to keep higher quality single nucleotide polymorphisms (SNPs; call-rate ≥ 0.98, MAF ≥ 0.01, and Hardy Weinberg equilibrium *P* ≥ 1 × 10^−6^) and removed variants with known type 2 diabetes associations and located in known high linkage disequilibrium (LD) regions. This resulted in 101,094 (~190K analysis) and 77,174 (~440K analysis) variants. Then we used PLINK^62,63^ to prune this set of variants to independent SNPs (--indep-pairwise 1000kb 1 0.2), resulting in 46,423 (~190K analysis) and 39,138 (~440K analysis) variants for downstream sample QC where independent genotypes are required.

### Data QC: Ancestry inference

To infer sample ancestry, we first merged their genotypes with reference genotypes on a set of 5,835 known ancestry informative SNPs. The reference genotypes used were 2,504 1000 Genomes Phase 3 Version 5 samples^64,65^. The merged data consisted of 5,741 (~190K analysis) and 3,511 (~440K analysis) variants. Principal components (PCs) were computed using FlashPCA2^66^ (**Supplementary Fig. 7)** and used as features to cluster the samples into one of five major population groups.

We applied the K-nearest neighbor (KNN) method^67^ to infer the ancestry of the study samples using the “knn” function from the “class” package in R^68^. We first trained the model using the 1000 Genomes samples and their known ancestry labels. Then, the K-nearest 1000 Genomes neighbors to the study samples were determined. The value of K was set to the floor of the square root of the sample size. The maximum label count of the K-nearest neighbors determined the ancestry assignment for each study sample. We applied the same algorithm to the first 3, first 4, first 5, ..., first 20 PCs of ancestry and settled on the highest count prediction among the 18 iterations. All samples were assigned to one of the five groups with no outliers (**Supplementary Fig. 7**; **Supplementary Table 16**).

### Data QC: Sample QC

To identify samples with obvious genotyping problems, we assessed both datasets for sample genotype missingness (< 0.5) and found that all samples passed the threshold for inclusion in our sample QC pipeline.

First, we assessed duplicates and cryptic relatedness. Sample pair kinship coefficients were determined using KING v2.2.8 relationship inference software^69^ (~190K analysis), which offers a robust algorithm for relationship inference under population stratification, and PLINK2^63^ v2.3a (~440K analysis). PLINK2 implements the same approach as KING but ran more efficiently on the much larger dataset. We identified 27 sample pairs as duplicates in the data (kinship > 0.4). Upon manual inspection, if the clinical data (e.g., date of birth) for any of the duplicate pairs was nearly identical, the sample with the higher call rate was reinstated. If the clinical data did not match or a manual inspection was not performed, both samples were removed. In addition to identifying duplicate samples, we checked for samples that exhibited kinship values indicating a second degree relative or higher relationship with 10 or more other samples. We found no samples that exhibited this cryptic relatedness.

Second, we conducted sex chromosome check. Each batch of data was checked for genotypic / clinical data agreement for sex. We used the “impute_sex” method in Hail^70^ v0.2.95 to calculate the inbreeding coefficient on the X chromosome genotypes. The default settings of below 0.2 (female) and above 0.8 (male) were applied to infer sex. 56 (~190K analysis) and 4 (~440K analysis) samples were flagged as a “PROBLEM” by Hail because it was unable to impute sex. This analysis did not identify any samples that had disagreement between genotypic and clinical sex; therefore, no samples were flagged for removal.

Third, we conducted sample outlier detection. Each sample was evaluated for inclusion in association tests based on 10 sample-by-variant metrics (**Supplementary Table 17a**), calculated using Hail. For the metrics n_called and call_rate, only samples below the mean were filtered. Due to possible population substructure, the sample metrics exhibited some multi-modality in their distributions. To evaluate more normally distributed data, we calculated principal component adjusted residuals of the metrics (PCARMs) using the top 10 principal components. For outlier detection, we clustered the samples into Gaussian distributed subsets with respect to each PCARM using the software Klustakwik^71,72^. During this process, samples that did not fit into any Gaussian distributed set of samples were identified and flagged for removal.

In addition to outliers along individual sample metrics, we also identified samples that exhibited deviation from the norm across multiple metrics. To identify samples of this nature, we calculated principal components explaining 95% of the variation in 8 of the 10 PCARMs combined. The adjusted residuals for metrics “call_rate” and “n_called” are characterized by long tails that lead to the maximum value, which is not consistent with the other metrics. To avoid excessive flagging of samples with lower, yet still completely acceptable, call rates, these metrics were left out of principal component calculation.

All samples were clustered into Gaussian distributed subsets along the principal components of the PCARMs, again using Klustakwik^71,72^. This effectively removed any samples that were far enough outside the distribution on more than one PCARM, but not necessarily flagged as an outlier on any of the individual metrics alone. The distributions for each PCARM and any outliers (cluster = 1) found are shown in **Supplementary Fig. 8**. In summary, in the ~190K analysis, we removed 181 samples as outliers (**Supplementary Table 17b**). Among the remaining samples, only 189,947 samples of European ancestry with phenotypic values were used for association testing (51,582–189,787 samples across 55 phenotypes; **Supplementary Table 4**). In the ~440K analysis, we removed 188 samples as outliers (**Supplementary Table 17b**). Among the remaining samples, only 439,829 samples of European ancestry were used for association testing (111,917–439,829 across 55 phenotypes; **Supplementary Table 4**).

### Variant annotation

We annotated variants with the VEP^25^ v110.1, LOFTEE^13^ plug-in v1.0.3 (~190K analysis) and v1.0.4 (~440K analysis) and dbNSFP^26^ plug-in v4.4a. We selected the canonical annotations for each variant (PICK = 1).

### Mask construction: Previously employed masks

In the 234 shortlisted studies, 664 masks were employed. Among these, we removed 63 masks that did not have well-documented definitions and subsequently, 25 publications (**Supplementary Table 2a**).

Next, to replicate the remaining 601 masks, we harmonized them by translating the definitions into the closest equivalent in VEP. Since different publications used different reference databases, algorithms and tools to annotate variants, we made some adjustments as follows:

- If the studies used MAF from a reference database such as 1000 Genomes, gnomAD, etc. or from an internal cohort, we substituted it with MAF within the UK Biobank samples in our analyses.
- If the studies analyzed dichotomous traits and used MAF from only the control group, we substituted it with MAF within the UK Biobank samples used in our analyses.
- If PolyPhen2^15^ was employed without specifying which version, we included both PolyPhen2-HDIV and PolyPhen2-HVAR.
- If splice sites were included without further explanation, we included both splice donor variants and splice acceptor variants and excluded splice donor 5th base variants.
- Nonsynonymous with little explanation was considered missense.
- MAF less than or equal to a certain threshold was converted to MAF less than.

Among the 601 masks, we removed 141 masks that either included non-coding variants, only included synonymous variants (often used as negative controls) or could not be reconstructed using VEP annotations with reasonable minor adjustments (**Supplementary Table 2a**). Some examples include variants annotated as pathogenic to a specific trait based on experts’ opinions or certain databases, variants previously identified as causal, and variants located in constrained regions. Among the remaining 460 masks, duplicates (after VEP harmonization) were treated as one mask only, resulting in 298 unique masks and 154 unique masking strategies employed in 169 publications (**Supplementary Table 2c)**. Out of the 298 masks, 271 masks remained for further analyses after association testing (see below).

### Mask construction: New masks

We derived three types of new masks. First, we applied MAF < 1% and MAF < 0.1% thresholds to the 271 previously employed masks, resulting in 117 additional unique masks (some of the 271 masks already included these filters). Second, we systematically combined three MAF thresholds (all MAF, MAF < 1% and MAF < 0.1%) with high-confidence pLoF variants predicted by LOFTEE^13^ and missense variants predicted to be damaging by at least 25%, 50%, 75% and 90% of the 39 bioinformatic algorithms with rank scores in dbNSFP^26,73^ (original scores), resulting in 12 additional masks (**Supplementary Tables 2d, 6, 18a**). On average, the 21 algorithms that had categorical predictions for missense variants predicted 33% of the variants to be damaging (**Supplementary Table 18b**); therefore, we defined a variant to be damaging by an algorithm if its rank score was larger than 0.67.

Finally, because these bioinformatic algorithms produced highly correlated predictions, we also defined damaging scores based on “composite” annotations derived from either (a) principal component analysis^38^ (PCA) or (b) independent components analysis^74^ (ICA) of the bioinformatic algorithms, resulting in 24 additional masks (**Supplementary Tables 2d, 6, 18a**). We first imputed rank scores for missing variants using Iterative Imputer in scikit-learn^75^ and applied PCA on the rank scores. We then calculated the rank scores of variants for each PC. The original rank scores from 44 bioinformatic algorithms were all negatively correlated with MAF, suggesting that rarer variants were more likely to be damaging^1^ (**Supplementary Table 18a**). Therefore, if the PC scores were negatively correlated with the variants’ MAF (Pearson correlation; *P* < 0.01), we kept the rank scores of variants for each PC, and if the PC scores were positively correlated, we inverted the rank scores (**Supplementary Table 18c**). A variant was considered damaging by a PC if its rank score was greater than 0.67. A weighted average was then calculated based on the binary predictions of the PCs and their variance explained ratio output by PCA (PC scores; **Supplementary Table 18d**). A similar approach was done for ICA to generate IC scores for the variants (**Supplementary Table 18e**). Finally, we systematically combined three MAF thresholds (all MAF, MAF < 1% and MAF < 0.1%) with high-confidence pLoF variants predicted by LOFTEE and missense variants with PC scores or IC scores of at least 25%, 50%, 75% and 90%, resulting in 24 additional masks. The PC scores were strongly correlated with original scores (Pearson correlation coefficient = 0.83, *P* = 0) whereas IC scores were moderately correlated with both original scores (Pearson correlation coefficient = 0.28, *P* = 0) and PC scores (Pearson correlation coefficient = 0.2, *P* = 0). In total, we added 153 new masks to the analysis, resulting in 424 masks.

### Association testing: Single-variant tests

We conducted single-variant association tests for 55 phenotypes on 17,605,686 in 189,947 samples using REGENIE^76^ (v3.1.2) implemented in dig-loam pipeline^77^ (~190K analysis) and 21,500,124 variants in 439,829 samples using REGENIE^76^ (v3.2.5) implemented in the UK Biobank Research Analysis Platform (~440K analysis). For step 1, the following parameters were set: --bsize 200 –qt. For step 2, the following parameters were set: --minMAC 1 --bsize 200 –qt. We ran the association tests with sex, genotyping array, and the first 5 PCs as covariates.

The association results were used subsequently to determine gene-level associations driven by one single variant or by the aggregate burden of many variants, and to rerun the burden tests for significant associations conditioned on the common variants (MAF ≥ 1%) associated with each trait.

### Association testing: Gene-level tests

In the ~190K analysis, we conducted 16,445 gene-level burden tests for 298 previously employed masks and a synonymous mask as a negative control across 55 phenotypes using REGENIE^76^ (v3.1.2) implemented in dig-loam pipeline^77^ (16,445 = (298+1) × 55). For step 1, the following parameters were set: --bsize 200 –qt. For step 2, the following parameters were set: --minMAC 1 --aaf-bins 0.5,0.01,0.001 --bsize 200 --build-mask sum --qt --write-mask-snplist.

We ran the association tests with sex, genotyping array, and the first 5 PCs as covariates. We applied the 50%, 1%, and 0.1% MAF threshold for each test to (a) obtain significant associations driven by low-frequency and rare variants, and (b) generate association results for new masks derived from the 271 masks. We noted that the MAF filter in each mask was the overall MAF of the entire ~190K UK Biobank samples, whereas additional MAF filters in REGENIE were specific to each phenotype. However, the differences in the variants included in each mask were negligent and did not affect the gene-level association results.

To calibrate our results, we removed 21 masks consisting of no variants, and hence, produced no association results (**Supplementary Table 5a**). Next, we removed 6 highly inflated masks with an average lambda across 55 phenotypes > 1.2 (**Supplementary Table 5a**). Finally, we removed height as the association results for height were much more inflated than the other 54 phenotypes (**Supplementary Table 5b**). This resulted in 14,634 gene-level burden tests for 271 masks and 54 phenotypes (14,634 = 271 × 54). Among the genes tested, we removed those with cumulative MAC ≤ 10.

The significance threshold on a mask level was set at *P* < 2.5 × 10^−6^ and on a masking strategy level was set at *P* < 2.5 × 10^−6^/number of masks. Among the significant associations in each mask, we reran the burden test conditioned on common variants associated with each trait using LD clump function in PLINK2^63^ with the default parameter settings.

In the ~440K analysis, we conducted 864 gene-level burden tests similar to the ~190K analysis but using REGENIE^76^ (v3.2.5) in the UK Biobank RAP for 54 phenotypes and 16 masks in the recommended low-frequency and rare masking strategies (864 = 54 × 16; see below). To assess the association results produced by the recommended masks, we obtained the gene-level association results in European ancestry for 46 out of 54 phenotypes (**Supplementary Table 4**) available in three recent high-profile studies of the UK Biobank as follows:

- Results for “UK Biobank 470k (v5) public” for PMID: 34375979^30^ (only associations with *P* < 0.1 were available) accessed via the “https://azphewas.com” API.
- Results downloaded from “gs://ukbb-exome-public/500k/results/results.mt” for PMID: 36778668^23^.
- Results downloaded from “https://www.ebi.ac.uk/gwas/publications/34662886” for PMID: 34662886^22^.

Genomic inflation levels from PMID: 34662886^22^ and PMID: 36778668^23^ were similar to those observed in the ~440K analysis (**Supplementary Figs. 9a-c**).

### Selection of masking strategies: Previously employed masking strategies

For each of the total, low-frequency, and rare association analyses, we defined the “best” masking strategy previously employed as the masking strategy used in a past study that detected the largest number of significant associations. Similarly, we defined the “average” masking strategy previously employed as a proxy strategy that detected the average number of significant associations across all strategies used in past studies.

### Selection of masking strategies: Clustering methods

To improve upon the previously employed masking strategies and the brute-force 271-mask strategy, we attempted to identify redundant masks by applying PCA in scikit-learn^75^, using the binary inclusion of UK Biobank variants as features for each of the 271 masks (variant membership clustering). The first 9, 18, and 40 principal components (PCs) explained 90%, 95% and 99% of the total variance of the 271 masks (**Supplementary Table 9a**). We then applied k-means clustering in scikit-learn^75^ to cluster the masks based on the first 9 PCs. The cost function of k-means clustering reduced minimally after 10 clusters, suggesting that 10 was the optimal number of clusters (**Supplementary Fig. 3a**). Since there was substantial heterogeneity within each cluster (silhouette score = 0.51; **Supplementary Fig. 3b**), we further applied PCA and k-means to each cluster and identified 37 subclusters (variant MAF clustering; **Supplementary Table 9b**). In each of the clusters and subclusters, we selected the mask with the largest number of significant associations as the “representative” mask, resulting in a 10mask strategy and a 37-mask strategy for each of the total, low-frequency and rare association analyses (**Supplementary Tables 9c-d**).

### Selection of masking strategies: Greedy covering method

A comprehensive strategy to assess which masking strategies would yield the maximum number of significant associations after Bonferroni correction would be to compare all the possible 2^271^ combinations of 271 masks. Since it is computationally impossible to calculate the number of significant associations detected by such a large number of strategies, we developed a greedy covering method.

First, we applied a divide-and-conquer approach to group the masking strategies into subgroups consisting of the same number of masks, i.e., having the same size ***m*** (***m*** *∈* [1..271]), and hence, the same Bonferroni correction penalty. Next, to determine which masking strategy detected the largest number of significant associations (highest-yield strategy) within each subgroup of size ***m***, we applied a depth-first search as follows: (1) for each mask, obtain significant associations (*P* < 2.5 × 10^−6^/**m**), (2) order the masks based on the number of significant associations, (3) pick the mask with the largest number of significant associations, (4) remove the significant associations already detected by the selected mask from the remaining masks, (5) re-order the masks based on the number of significant associations, and (6) repeat steps 3–5 until ***m*** masks were selected. Finally, we compared the number of significant associations detected by the highest-yield strategies of all 271 subgroups and selected the overall highest-yield strategy. We defined this as the “optimal” masking strategy.

To examine the robustness of the greedy covering method, we conducted a cross-validation test for each of the total, low-frequency and rare association analyses. For each phenotype ***p***, we applied the greedy covering method to obtain the trait-specific optimal masking strategy which detected the maximum number of significant associations. Then, we applied this method to the other 53 traits altogether and compared the significant associations for ***p*** detected by the 53-trait optimal masking strategy to the maximum number of significant associations for ***p***. We found that on average, the 53-trait optimal masking strategies produced 87.7% (SD = 16.8%) as many total, 62% (SD = 23.7%) as many low-frequency, and 53.4% (SD = 26.1%) as many rare significant associations as the trait-specific optimal masking strategies (**Supplementary Fig. 4; Supplementary Table 11**). Outlier traits having 0 or 1 low-frequency or rare significant association detected by the 53-trait optimal masking strategies also had fewer than 10 maximum significant associations. Considering only traits with at least 10 maximum significant associations, the coverage improved (mean = 92.5%, SD = 5.4% for total associations; mean = 73.5%, SD = 11.6% for low-frequency associations; mean = 69.4%, SD = 12.3% for rare associations).

We then applied the greedy covering method to all 54 phenotypes combined. From 271 masks, the method produced trait-agnostic optimal 7-mask, 26-mask, and 26-mask strategies that maximized the number of total, low-frequency and rare significant associations. Notably, the number of masks in the optimal strategy for total associations were substantially smaller than those for low-frequency and rare associations. As we increased the number of masks in the greedy method, the number of significant associations removed due to increased Bonferroni correction exceeded the number of significant associations gained for the analysis of total associations sooner than it did for low-frequency and rare association analyses (**Supplementary Fig. 10a**). Specifically, we found that the 7-mask strategy consisted of many common variant (MAF ≥ 1%) associations detectable with only a few masks, and the comparatively small number of additional significant associations detected by adding more masks could not compensate for the loss of common variant associations from increased Bonferroni correction (**Supplementary Fig. 10b**). By contrast, the number of low-frequency and rare significant associations added with more masks was much more linear, outweighing the cost of increased Bonferroni correction (**Supplementary Table 12a**). Furthermore, as the number of masks increased, the marginal increase in Bonferroni correction penalty decreased, making it even easier to add more masks (**Supplementary Fig. 10c)**.

Interestingly, however, when we repeated the analysis for each trait individually, the opposite was typically true (**Supplementary Table 19**). For 47 out of 54 traits, the number of masks in the total optimal strategy was larger than or equal to the number in the low-frequency and rare optimal strategies. Consequently, while the sizes of the trait-specific optimal strategies for total associations (mean = 7.7, SD = 3.84) were comparable to that of the trait-agnostic 7-mask strategy, the sizes of the trait-specific low-frequency and rare optimal strategies were much smaller (between 1 and 13 masks) than those of the trait-agnostic 26-mask low-frequency and rare optimal strategies. This difference in behavior was due to the high overlap of trait-specific optimal strategies for total associations (mean of Jaccard similarity scores = 0.26, SD = 0.15) compared to a near-zero overlap for low-frequency associations (mean of Jaccard similarity scores = 0.03, SD = 0.05) and rare associations (mean of Jaccard similarity scores = 0.02, SD = 0.05). For example, for total associations, the optimal strategy specific to alanine aminotransferase (ALT) captured 92.98% as many trunk lean (fat-free) mass (TrunkLM) associations detected by the TrunkLM-specific optimal strategy; however, for low-frequency associations, the ALT-specific optimal strategy captured only 37.04% as many associations found by the TrunkLM-specific optimal strategy. These results suggest that the ability of masks to detect most low-frequency and rare significant associations is much more trait dependent than their ability to detect most total significant associations, and consequently that – to maximize power – trait-agnostic masking strategies for low-frequency and rare associations must be much larger than trait-agnostic strategies for total associations.

Finally, we applied the greedy covering method to three nested sub-groups of 424 masks (271 previously employed masks + 153 new masks; see above). These sub-groups corresponded with three types of associations: rare (211 masks), low-frequency (352 masks, including rare masks), and total (424 masks, including rare and low-frequency masks) (**Supplementary Table 6**). From 424 masks, the method produced trait-agnostic optimal 6-mask, 22-mask, and 35-mask strategies that maximized the number of total, low-frequency and rare significant associations (**Supplementary Table 12b**).

### Gene set analyses

In the ~440K analysis, for each gene/trait pair, we applied a previously described method to aggregate the mask-level p-values produced by the recommended rare 8-mask strategy into one aggregate p-value^21^. We then conducted gene set enrichment analysis for 4,203 gene sets from the Mouse Genome Informatics database^39^ as previously described^21^. We then applied FDR correction and considered a gene set to be significantly enriched for a trait if the FDR-adjusted *P* < 0.05.

## Figures and Tables

**Table 1.** Mask definition and the number of significant associations detected by each mask in the recommended masking strategy. See **Supplementary Table 2d** for more details on mask definitions.

Association type	Mask	Mask definition	Number of significant associations	Number of significant associations added to the masking strategy	Number of significant associations cumulated in the masking strategy
Total associations	23633568_m1	stop_gained | frameshift | (missense & (SIFT_pred_D | Polyphen2_HVAR_pred_D_or_P | Polyphen2_HDIV_pred_D_or_P))	2164	2164	2164
Total associations	32141622_m7	stop_gained | essential_splice | frameshift | splice_region_variant	1320	1021	3185
Total associations	new_damaging_ic25	(LoF_HC | (missense & combo_ic25))	2133	859	4044
Total associations	30828346_m1	(missense | synonymous | stop_gained | stop_lost) & maf10	1370	591	4635
Total associations	new_damaging_og25	(LoF_HC | (missense & combo_og25))	1273	186	4821
Total associations	32141622_m4	indels	201	131	4952
Low-frequency associations	new_damaging_og25.0_01	(LoF_HC | (missense & combo_og25)) & maf1	349	349	349
Low-frequency associations	32853339_m1	(IMPACT_HIGH | ClinVar_pred_P_or_LP) & maf1	245	99	448
Low-frequency associations	29177435_m1	(incomplete_terminal_codon | start_retained | stop retained | synonymous | IMPACT MODERATE | IMPACT_HIGH) & maf1	258	84	532
Low-frequency associations	29378355_m1.0_01	(stop_gained | missense | essential_splice) & maf1 & maf_gt_0_5	148	58	590
Low-frequency associations	36327219_m3	(IMPACT_HIGH | (indels & IMPACT_MODERATE) | (missense & Polyphen2_HDIV_pred_D & Polyphen2_HVAR_pred_D & LRT_pred_D & MutationTaster_pred_D_or_A & SIFT_pred_D)) & maf1	324	49	639
Low-frequency associations	new_damaging_og50.0_01	(LoF_HC | (missense & combo_og50)) & maf1	309	31	670
Low-frequency associations	24507775_m6.0_01	(stop_gained | stop_lost | essential_splice | Polyphen2_HDIV_pred_D | Polyphen2_HVAR_pred_D) & maf1	304	29	699
Low-frequency associations	32141622_m7.0_01	(stop_gained | essential_splice | frameshift | splice_region_variant) & maf1	140	19	718
Rare associations	31383942_m4	(stop_gained | stop_lost | frameshift | essential_splice | (missense & REVEL_score_0_55)) & maf0_1	232	232	232
Rare associations	37348876_m8	(CADD_phred_20 | LoF_HC) & maf0_1	225	64	296
Rare associations	31383942_m10	(stop_gained | stop_lost | frameshift | essential_splice | ClinVar_pred_P_or_LP) & maf0_1	207	53	349
Rare associations	31118516_m5.0_001	(LoF_HC | (Polyphen2_HDIV_pred_D & Polyphen2_HVAR_pred_D & SIFT_pred_D & LRT_pred_D & MutationTaster_pred_D_or_A)) & maf0_1	210	24	373
Rare associations	36411364_m4.0_001	(LoF_HC | (missense & REVEL_score_0_75)) & maf0_1	220	15	388
Rare associations	34183866_m1	(stop_gained | essential_splice | frameshift | indels | (missense & Polyphen2_HDIV_pred_D)) & maf0_01	162	13	401
Rare associations	30269813_m4	(stop_gained | essential_splice | frameshift | indels | (missense & (Polyphen2_HDIV_pred_D | Polyphen2_HVAR_pred_D))) & maf0_1	223	11	412
Rare associations	34216101_m3.0_001	(Polyphen2_HDIV_pred_P | Polyphen2_HVAR_pred_P) & maf0_1	47	10	422

## Data Availability

All genotyping data for the ~190K analysis was accessed from the UK Biobank (Application ID: 41189). All genotyping data for the ~440K analysis is accessible on the UK Biobank Research Analysis Platform (ukbiobank.ac.uk/enable-your-research/research-analysis-platform). Gene-level burden results from the recommended low-frequency and rare masking strategies for 54 continuous phenotypes are publicly accessible on a web browser developed using Bring Your Own Results services (BYOR; byor.science) provided by the Knowledge Portal Network at the Broad Institute of MIT and Harvard (hugeamp.org:8000/research.html?pageid=trang_mask_paper_home). The variant group files for the recommended masking strategies from the exome sequences of ~440K samples of European ancestry are also available for multiple formats on the same web browser (hugeamp.org:8000/research.html?pageid=trang_mask_paper_home).
